# Sexual Dimorphism in Chronic Hepatitis B Virus (HBV) Infection: Evidence to Inform Elimination Efforts

**DOI:** 10.12688/wellcomeopenres.17601.1

**Published:** 2022-01-31

**Authors:** Robin Brown, Philip Goulder, Philippa C. Matthews

**Affiliations:** 1 Harris Manchester College, University of Oxford, Oxford, Oxon, OX1 3TD, UK; 2 Department of Paediatrics, University of Oxford, Oxford, Oxon, OX1 3SY, UK; 3 The Francis Crick Institute, London, London, NW1 1AT, UK; 4 Division of Infection and Immunity, University College London, London, London, WC1E 6BT, UK; 5 Department of Infectious Diseases, , University College London Hospital, London, London, NW1 2BU, UK

**Keywords:** hepatitis, HBV, sex, gender, oestrogen, testosterone, androgen, cancer, hepatocellular carcinoma, gender, stigma, epidemiology, outcome, treatment, dimorphism

## Abstract

Sexual dimorphism in infectious diseases refers to the different infection susceptibilities and outcomes between males and females, and has been described for many pathogens, including hepatitis B virus (HBV) infection. HBV is a substantial global health problem, with close to 300 million people infected, and accounting for a million deaths each year, with an urgent need for enhanced interventions to support progress towards elimination goals. Sexual dimorphism has a strong influence in HBV infection, with males more likely to be exposed, to develop chronic infection, and to suffer from complications including cirrhosis and hepatocellular carcinoma (HCC) compared to females. Different outcomes are driven by differential immune responses, sexual dimorphism of the liver, and androgen response elements in the HBV genome. The impact of sex may also vary with age, with changes at puberty and influences of menarche, pregnancy and menopause in females. In addition, gender has complex influences on education, beliefs, behaviour and access to / engagement with healthcare services, which may contribute to differences in diagnosis and treatment. Interplay between these complex factors, alongside other attributes of host, virus and the environment, accounts for different outcomes of infection. However, gaps remain in our understanding of sexual dimorphism in HBV, and little effort has previously been made to harness this knowledge for translational gains. In this review, we assimilate human and animal data to consider the mechanism, outcomes and impact of sexual dimorphism, considering how these insights can be used to inform advances in surveillance, treatment and prevention for HBV infection.

## 1. Introduction

Chronic infection with hepatitis B virus (HBV) is estimated to affect 257 million people ^1^ and accounts for an increasing burden of the 1.34 million yearly deaths due to viral hepatitis ^2, 3^. United Nations Sustainable Development Goals (SDGs) underpin ambitious targets for the elimination of hepatitis viruses, with the Global Health Sector Strategy setting out aims to reduce new infections by 90% and mortality by 65% by 2030 ^4^. Improving our understanding of the natural history of chronic HBV infection (CHB) is central to inform progress through enhanced evidence-based treatment and prevention of liver disease ^5^.

Sex, defined as the biological characteristics that differ between males and females ^6^, accounts for significant immunological differences leading to disparities in outcomes for a variety of infectious diseases ^6, 7^, termed ‘sexual dimorphism’. The effect of host sex on outcomes of infection is complex and multifactorial, influenced by genetics, hormones, and environmental exposures ^7^, with trade-offs between protective immune responses (leading to clearance or control of infection) and immunopathology (associated with increased severity and duration of disease). Gender, as a societal and behavioural construct, also plays a role through its influence on perceptions, behaviour, and access to healthcare.

Among chronic viral infections, HIV exemplifies this complex picture, whereby females typically have lower titres of human immunodeficiency virus (HIV) RNA than males ^8^ with a 5-fold higher frequency of elite control ^9^. However, females also show increased immunopathology associated with an elevated risk of developing AIDS compared to males with the same HIV RNA levels ^6^, and a greater susceptibility to infection, with both a biological and societal basis ^10, 11^. UNAIDS data for 10–19 year olds in 2019 reported 33,000 adolescent girls becoming HIV infected compared to 4,200 boys ^12^. Hepatitis C virus (HCV) has a higher prevalence in males, with females being more likely to clear the virus and also suffering fewer complications ^13, 14^. Sexual dimorphism in HBV has been less rigorously studied, but was first described by Baruch Blumberg in 1972 ^15^, nine years after his discovery of ‘Australia antigen’ (now termed Hepatitis B surface antigen, HBsAg), underpinning subsequent consistent observations of an increased risk of chronic infection and its complications among males compared to females.

HBV is a partially double-stranded DNA virus which archives itself in the nucleus of hepatocytes as a covalently closed circular (ccc)-DNA ‘mini-chromosome’ accounting for persistent chronic infection, that can lead to inflammatory liver disease, fibrosis, cirrhosis and hepatocellular carcinoma (HCC) ^16, 17^. Viral factors, the liver micro-environment, and host attributes all contribute to sexual dimorphism in CHB, as previously reviewed ^13, 18, 19^. However, HBV infection has been relatively neglected by research, clinical care, public health interventions, and advocacy ^20^, and females are specifically under-represented by translational research ^21, 22^.

In this review, we discuss sexual dimorphism in CHB, considering the relevance of sex vs. gender, and the specific influence of menarche, pregnancy and menopause in females. We consider how an improved understanding of differential outcomes between males and females may (i) underpin new insights into the pathophysiology of liver disease, (ii) improve patient stratification for surveillance and treatment, (iii) inform new approaches to personalised therapy, and (iv) optimise public health measures and resource allocation. This would support advances towards elimination goals.

## 2. Epidemiology of HBV infection according to sex

Blumberg’s 1972 metanalysis of HBV prevalence included studies in 23 disparate populations, including cohorts with leprosy, trisomy 21, and renal dialysis patients. HBsAg carriage was more prevalent in males in 22/23 of the populations studied ^15^. The male:female ratio ranged from 3.58 to 0.855 and, interestingly, was found to be greatest in the 0–19 age groups in all but one of the studies where age stratification was possible.

More recently, the male:female sex ratio in CHB has been reported as 1.2 in asymptomatic carriers, increasing to 6.3 in chronic liver disease and 9.8 in HCC ^23^, but these estimates vary between settings. Selected studies reporting the prevalence of HBsAg in males and females are presented in Table 1, with odds ratio of infection as high as 1.9 in males (95% CI, 1.2–3.2) ^24^.

**Table 1.** Exemplar studies reporting HBsAg prevalence in males and females.

Study setting	Number in study	Seroprevalence data	Citation
Blood donors, Crete	65,219	HBsAg 0.41% in males and 0.28% in females; OR for males 1.9 (95% CI, 1.2-3.2)	Koulentaki, M. *et al.* ^24^
Migrants and refugees, Southern Italy	1,212	HBsAg seroprevalence 9.6% overall. OR for males 1.8 (95% CI, 1.3-2.5)	Coppola, N. *et al.* ^31^
Adults age 35-44, general population, Taiwan	45,035	Seroprevalence 17.8% in males vs. 13.2% in females (p < 0.001); difference diminished in age >60. HBsAg male to female prevalence ratio 1.49	Tsay, P. K. *et al.* ^32^
Meta-analysis of 27 studies, China	5,422,405	HBsAg prevalence of 5.8% in males (95% CI:5.53– 6.24%) and 5.05% in females (95% CI:4.56–5.88%)	Wang, H. *et al.* ^33^
Meta-analysis of 20 studies in diverse groups, Pakistan	81,755	HBsAg prevalence for general population 2.71% (95% CI 1.74 to 4.21). Three times more prevalent in males than females (OR not formally presented).	Khan, N. U. *et al.* ^34^
Comparison of pre-vaccine and post-vaccine studies in Australian Aboriginal and Torres Strait Islander People from 36 studies	501,622 *	Decrease in the pooled prevalence of HBsAg over time among women (from 4.2% to 2.2%) and men (from 17.5% to 3.5%). No OR reported.	Graham, S *et al.* ^35^

95% CI – 95% confidence interval. HBsAg – Hepatitis B virus surface antigen * Total number tested is not reported in this study, so denominator calculated from study meta-data, but we cannot exclude the possibility of some population groups being represented more than once.

None of the metanalyses we identified ( Table 1) could use data from all of the studies identified due to poor and inconsistent reporting of disaggregated sex data. They also suffered from substantial heterogeneity between studies and potential bias due to inclusion of specific high-risk populations. Similarly, large scale HBV metanalyses of HBV prevalence from Ethiopia and Burkina Faso were unable to analyse a sex ratio from their metadata ^25, 26^. This highlights a consistent difficulty that currently inhibits robust meta-analysis to more accurately delineate sex differences in HBV prevalence.

**Figure 1.**
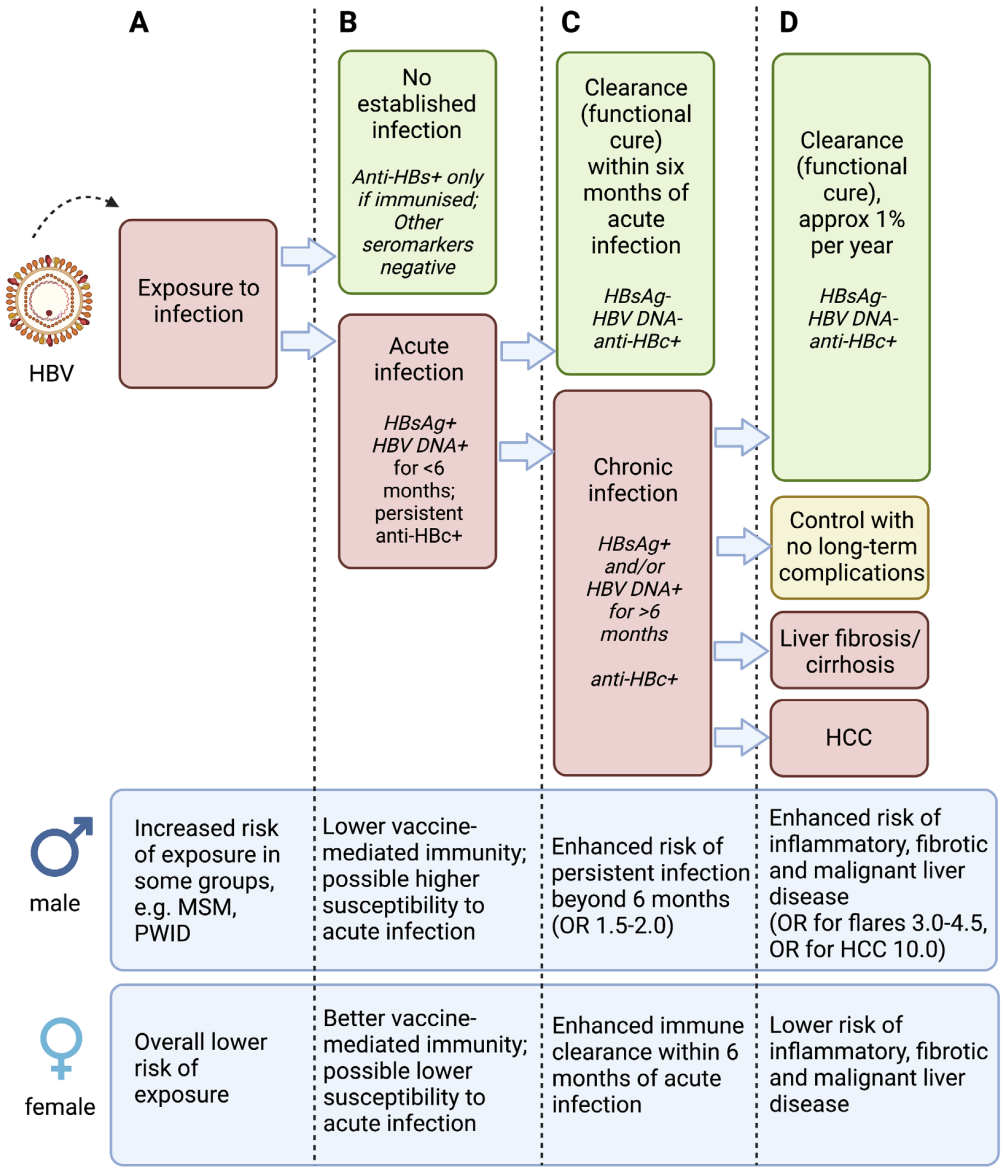
Schematic to illustrate phases of hepatitis B virus (HBV) infection and relevant sex differences. Infection is considered according to exposure to the virus (column A), acute infection (column B), chronic infection (column C) and liver disease (column D), with data for males and females presented at the bottom of each column. Anti-HBs – antibody to HBV surface antigen; HBsAg – Hepatitis B virus surface antigen; Anti-HBc – antibody to HBV core protein; HCC – hepatocellular carcinoma; MSM – men who have sex with men; PWID – people who inject drugs. OR (odds ratio) for males is presented based on females as the reference group. There is varied evidence for the specific observations presented in this figure, and the magnitude of increased risk in males varies between populations and settings. Figure created with BioRender.com, with licence to publish.

A key question to be addressed is whether CHB prevalence is lower in females due to less exposure events, lower susceptibility to acute infection, or enhanced clearance compared to males, or a combination of all of these ( Figure 1, columns A, B and C respectively). In part, this could be assessed by comparing the prevalence of anti-HBc antibodies (indicating exposure, with or without clearance) and HBsAg (indicating current infection) between males and females. In a cohort of 31,990 blood donors from Crete, HBsAg prevalence was 0.41% in males vs 0.28% in females (OR 1.98, 95% CI 1.2–3.2), and exposure was also higher in males than females, with anti-HBc prevalence of 9.16% vs 5.86% respectively ^24^. A large-scale metanalysis in 2019 identified no significant difference in spontaneous HBsAg seroclearance between females and males ^27^. However, there was substantial heterogeneity between studies and HBsAg clearance is an uncommon event ^28^, so the study may have been underpowered to detect a sex influence.

The epidemiological literature for HBV suffers from over-representation of specific populations that are subject to sex or gender bias. For example, certain defined populations, such as healthcare workers, pregnant women, waste handlers, men who have sex with men, people who inject drugs, and sex workers are often studied ^29, 30^. The increased prevalence of HBsAg in males may reflect a complex combination of exposure risk, response to acute infection, and outcomes in chronic infection. More epidemiological data are needed, disaggregated by age, sex and disease characteristics, in order to determine local requirements for provision of public health and clinical services, and to build a clear picture of the global burden of disease.

## 3. Relationship between sex and morbidity and mortality in chronic HBV infection

### 3.1 Sexual dimorphism in liver disease

The liver is a highly sexually differentiated organ, with up to 70% of genes showing differences in expression between male and female mice ^19, 36^. In humans, females are relatively protected from chronic liver fibrosis, regardless of aetiology, including lower rates of NAFLD/NASH ^37^, a lower rate of cirrhosis and liver transplant ^38^ and a lower risk of hospitalisation and death from cirrhosis ^39^ compared to males. Pre-menopausal females with CHB are at a lower risk of chronic liver disease compared to males, as exemplified by a cohort of 672 patients in Switzerland, in which liver-related outcomes were significantly less common in females (OR 0.35, 95% CI 0.20–0.60) ^40^. Similarly, in a Canadian study of nearly 6000 patients with HBV infection, male sex was an independent predictor of advanced liver fibrosis ^41^. There is increasing recognition that diabetes and metabolic disease are associated with worse outcomes in HBV infection ^42^, which may be linked to increased disease in males, as differences in fat biology and metabolism differ by sex ^43^. Metabolic-associated fatty liver disease is also significantly more common in males, with a loss of protection in post-menopausal females ^44^.

### 3.2 Hepatocellular carcinoma (HCC)

**Figure 2.**
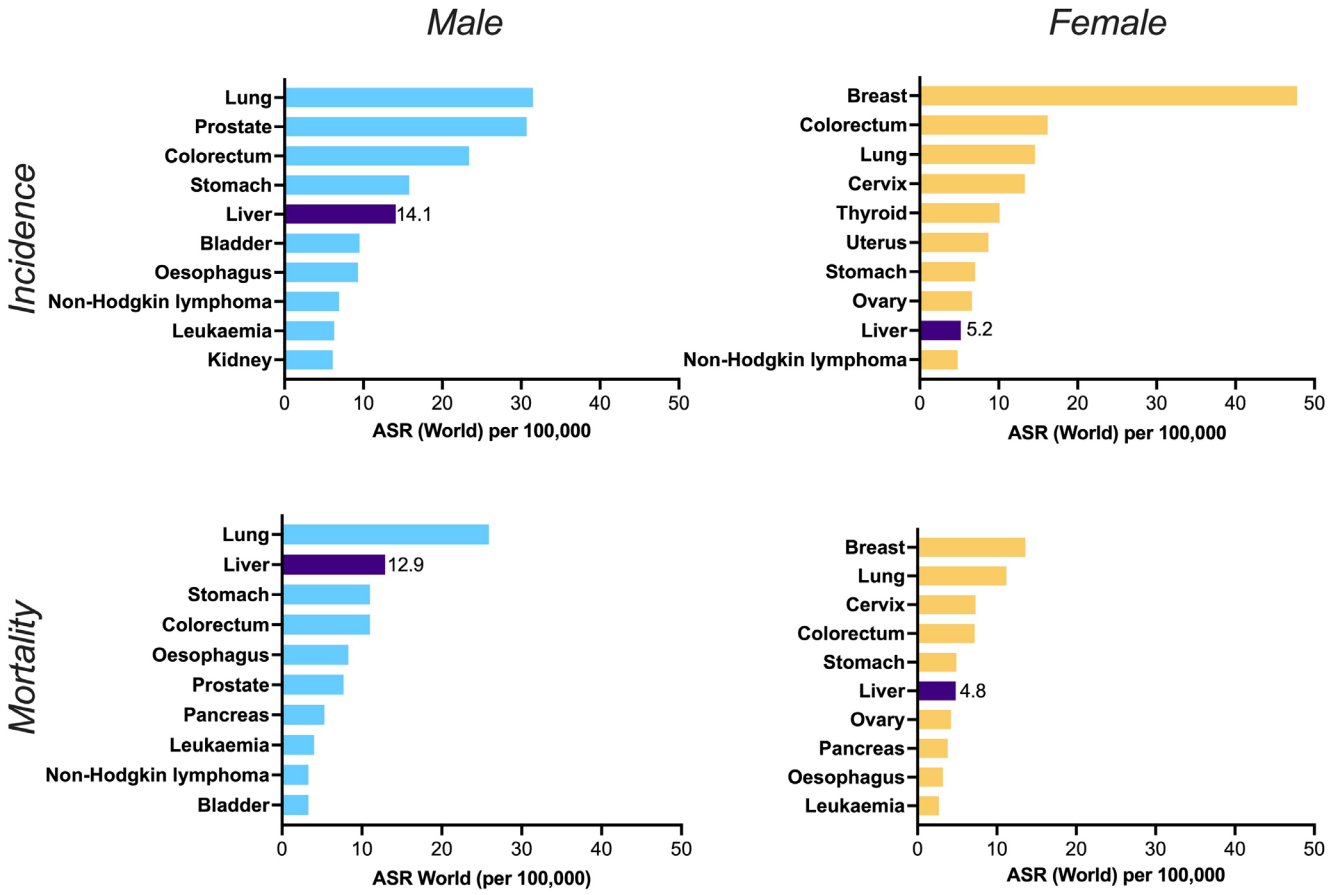
Worldwide incidence (top panels) and mortality (bottom panels) of top ten cancers in males (left panels) and females (right panels). ASR = age standardised rate. Liver cancer is shown in the dark purple bars, alongside the point estimate for ASR in each case, illustrating its place in the top five cancers for incidence and mortality in males, and the top ten for females. Data source: GLOBOCAN 2020 and the Global Cancer Observatory ( http://gco.iarc.fr). Primary liver cancer incidence among men and women is increasing sharply over time, in contrast to the declines for other cancers ^56^ and over half of this global burden is attributable to HBV infection ^45^. Figure created in GraphPad Prism.

HBV is the single biggest aetiological agent of liver cancer worldwide, causing over half of all cases (point estimate 56%, 95% CI: 52-60), but with substantial regional variation such that in parts of sub-Saharan Africa and Eastern Asia it is responsible for at least 2/3 of cases ^45^, and far more in some settings. Overall, the incidence and mortality of liver cancer is higher in men than in women ( Figure 2). A Global Burden of Disease study estimates annual incidence of primary liver cancer at 591,000 in males vs. 264,000 in females (ratio 2.2); among these, HBV-related HCC was estimated to have caused 203,000 cases in men and 70,000 in women (ratio 2.9) ^16^. In specific studies, male:female ratios for HCC are between 2:1 and 9:1. This wide variability is at least partly accounted for by the prevalence of CHB ^46^, as in settings in which HBV accounts for a high prevalence of HCC, the male:female ratios are typically high ^47^, for example in Senegal, where HBV accounted for almost 70% of HCC cases, the sex ratio was 6.6 ^48^, and in Vietnam, where >80% of HCC was HBV-associated, the ratio was 8.9:1 ^49^. HCC outcomes are worse when the aetiology is HBV ^50^, and in males compared to females ^51^.

These observations in humans are corroborated by evidence from animal models. Following exposure to high dose carcinogens such as diethylnitrosamine (DEN), liver inflammation and proliferation are more pronounced in male mice ^52^, and males exhibit a higher rate of tumorigenesis ^53^, with cancers that progress more rapidly, underlining male susceptibility to both the development and progression of liver disease. 

### 3.3 Flares of liver inflammation and acute-on-chronic liver failure

Flares of liver inflammation in HBV infection can be a consequence of the immune response targeting infected hepatocytes (leading to the favourable outcome of HBsAg clearance), or can represent organ damage (with adverse outcomes including liver failure, long-term risk of fibrosis/cirrhosis, and death) ^54^. Spontaneous flares of liver inflammation have been reported more commonly in males. For example, in a study of more than 1500 ethnically diverse adults followed over five years by the Hepatitis B Research Network in the US and Canada, flares were significantly commoner in males (OR 3.02; 95% CI: 1.59-5.74) ^55^. Smaller CHB cohorts corroborate this finding, with more flares in males in a study of 217 asymptomatic HBeAg-negative patients (OR 4.5; 95% CI: 1.5-20.8) ^57^ and in 386 patients followed up in China to identify exacerbations ^58^. In patients undergoing HBeAg seroconversion, male sex was again a significant risk factor for ALT flares ^59^, and likewise in a study exploring flares following treatment withdrawal ^60^. A prediction model for mortality in patients with acute on chronic liver failure in the setting of HBV infection includes male sex as a predisposing risk factor, with a view to informing improvements in stratified care ^61^.

Based on existing data, it is not possible to determine whether hepatic flares in males are linked to immune control or to progressive organ damage, although given other evidence of enhanced pathology in males it is natural to hypothesise the latter. More data are needed to explore these observations, as liver flares are likely to be under-reported, and are clearly linked to other host and viral factors (HBV treatment, immunosuppression, HBV genotype, age, viral load (VL)) making it complex to disaggregate the overall influence of sex ^54, 55^.

## 4: Mechanistic influence of sex in liver disease associated with HBV infection

### 4.1 Immunological differences between males and females causing differential outcomes of infection and vaccination

Females typically have higher magnitude innate, humoral and cytotoxic responses in response to infection, broadly reflecting an immunostimulatory effect of oestrogen ^6, 7^, in contrast to the suppressive effect of androgens in males. Across a range of infectious diseases, this potentially explains the increased severity and/or duration of illness in males compared to females ^62, 63^, and enhanced vaccine responses in females (discussed in further detail below). Sex dimorphism may be associated with differential expression of sex hormone receptors by immune cells (lymphocytes, monocytes and dendritic cells) and by hepatocytes. Chemokine and cytokine profiles also differ ^64– 66^, with androgens more typically associated with anti-inflammatory cytokines. Sex-specific differences in IL-6 levels have been associated with HCC, with elevated levels in males leading to activation of AR, while oestrogen inhibits IL-6 production in females ^67, 68^.

Toll-like receptor 7 (TLR-7) is encoded on the X chromosome, one copy of which is inactivated in females to render TLR-7 expression equivalent to that in males. TLR-7 stimulation activates plasmacytoid dendritic cells, stimulating production of type I interferons and thus promoting T and B cell responses ^69^, explaining why TLR-7 expression has been negatively correlated with HBV DNA VL ^70^. Escape of the second copy of TLR7 from inactivation can increase TLR-7 expression in females ^6, 71^. Furthermore, a SNP in the TLR-7 gene (rs179008) in has been associated with protection from chronic infection in females (but not in males) ^72^. Epigenetic modifications, and female X-chromosome mosaicism, may further contribute to this immunological advantage in females.

### 4.2 Sexual dimorphism in HBV vaccine responses

Males and females respond differently to HBV vaccination, with females mounting higher antibody titres than males ^73, 74^, and men more likely to be vaccine ‘non-responders’ (particularly in older age groups). For example, an Italian study reports that girls vaccinated after the age of 1 year mount a 1.2-fold higher median antibody titre than boys ^73^. This is in line with trends reported for other childhood vaccines, with females producing increased antibody titres and longer term durability of response ^74^. These effects may be due to the enhanced TLR-7 response in females, including a more inflammatory response to vaccine adjuvants. HBV vaccination in mice also demonstrates a higher rate of seroconversion in females, higher titres of anti-HBs antibody, higher magnitude T cell responses, and superior immunological memory ^75^. It is uncertain to what extent these differences are significant to the role of the HBV vaccine in supporting elimination efforts, but they illustrate fundamental differences in the quality and quantity of the immune response, and may account for some degree of enhanced male susceptibility, although this is difficult to quantify. Despite the sex differences in vaccine response, vaccination may blunt the overall difference between males and females. Thus, newer studies of younger subjects, in which the prevalence of vaccine-mediated protection is high, may be less powered to detect sex differences compared to pre-vaccine cohorts.

### 4.3 Interaction between sex hormones and the HBV replication cycle

**Figure 3.**
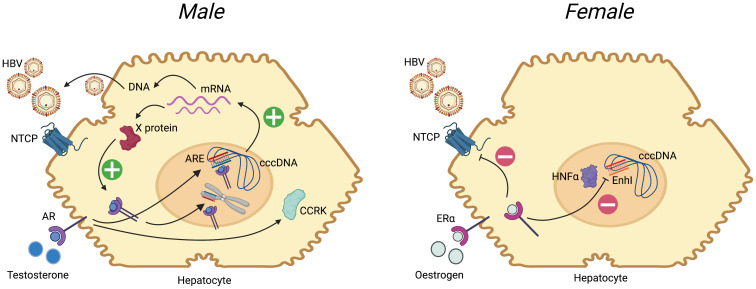
Summary of the influence of sex hormones on the HBV replication cycle in males and females. Following entry to the hepatocyte, mediated primarily by interaction with the sodium taurocholate co-transporter polypeptide (NTCP), HBV is trafficked to the nucleus. Host machinery archives the viral genome in the form of a stable covalently closed circular (ccc)-DNA molecule, which is the template for generation of mRNA species. mRNA is translated into new viral proteins (X, surface, capsid and polymerase), and reverse transcribed to DNA. ( **A**) In males, binding of testosterone to the androgen receptor (AR, a member of the steroid hormone nuclear receptor family) leads to dimerization of the receptor, which then binds to androgen response elements (ARE) in the HBV genome (both in cccDNA and integrated into host DNA). This promotes generation of mRNA species, leading to increased protein production, and pgRNA (pre-genomic RNA) which undergoes reverse transcription to DNA to generate new relaxed-circular DNA genomes. Increase in X protein feeds back on the androgen-AR complex in a positive feedback loop. Androgen-ARE complexes also stimulate cell cycle-related kinases (CCRK) stimulating proliferation. IL-6 is elevated in HCC and leads to activation of AR ^18^. ( **B**) In females, there is evidence that binding of oestrogen to oestrogen receptor alpha (ER-α) leads to down-regulation of the NTCP cell surface protein ^19^. Furthermore ER-α inhibits the binding of hepatocyte nuclear factor 4-alpha (HNF-α) to the Enhancer I (EnhI) region of the HBV genome, reducing mRNA production ^80^ (ref). In both panels, plus signs indicate stimulation/positive feedback, negative signs indicate blockade. For further details, see reviews ^13, 18, 19, 79^. Figure created with BioRender.com, with licence to publish.

HBV has a complex replication cycle, reviewed elsewhere ^76, 77^. The virus has numerous complex interactions with host cell proteins, and by integration into the host genome can also influence fundamental components of the cell cycle. The viral genome also has a direct influence through sex hormone response elements ^18^, summarised in Figure 3. A pathway to oncogenesis in males occurs through involvement of the androgen receptor (AR) signalling pathway, which is common to cancer evolution caused by diverse viruses including HBV, EBV, HTLV-1, HHV8 and HPV ^78^, and is also associated with the evolution of prostate cancer ^79^. In HBV infection, AR stimulation is associated with increased expression of all four HBV mRNAs, via two androgen response elements (ARE) in the enhancer 1 (EnhI) region of the HBV genome ^81^, leading to an upregulation in production of HBV proteins and DNA. In a mouse liver cell line, HB X protein increases AR activity in a dose-dependent manner ^82, 83^. Thus, a positive feedback loop operates between androgen exposure and X protein, which may explain the higher VL in males ^18, 84^ ( Figure 3). In human CHB infection, higher serum testosterone levels have been correlated with a higher incidence of HCC ^85^, consistent with the male/female ratio in HCC. In mouse models, interleukin-6 (IL-6) is elevated in male HCC, leading to activation of signal transduction and activator of transcription 3 (STAT3), which upregulates AR ^86^, and IL-6 has been suggested as a biomarker associated with worse outcomes in human HCC ^87^.

In contrast to androgens, oestrogens may actively suppress HBV replication. Activation of the oestrogen receptor alpha (Erα) can suppress HBV mRNA transcription by reducing expression of the transcription factor hepatocyte nuclear factor 4-alpha (HNFα). In a transgenic mouse model, HNFα increases HBV transcription by binding the enhancer I (EnhI) region of the HBV genome ^80^. Deletion of Erα in female mice removes the protective influence of oestrogen against hepatocarcinogenesis; relative to wild-type these knock-out animals had a nine-fold increased risk of tumorigenesis on exposure to carcinogens ^88^. In cell culture experiments, estradiol reduces expression of the hepatocyte surface protein NTCP (sodium taurocholate co-transporting polypeptide), the main entry receptor for HBV ^89, 90^, which is another possible mechanism for protection in females ^19^. Whether this mechanism is important in humans remains to be determined, but certain Erα polymorphisms have also been shown to increase the risk of acute liver failure in patients with HBV ^91^.

A longitudinal study of 4155 HBsAg positive individuals aged 30–64 years in the REVEAL-HBV study reported significantly higher HBV DNA VL in males (p<0.001); higher viraemia was associated with a greater risk of both HCC and cirrhosis ^84, 92^. In mouse studies, males have higher HBV VL and HBsAg levels ^93, 94^ compared to females ^81, 95– 97^. These effects are more pronounced after puberty, with VL lowered by orchidectomy in male animals and restored by androgen supplementation ^81, 93^. Conversely, VL is increased by oophorectomy in females ^80^. Both VL and HBsAg are sensitive to androgen receptor mutations, confirming the influence of this pathway in mediating the phenotype ^98^.

### 4.4 The influence of sex on response to treatment

Sex is also a predictor of response to antiviral therapy. Female sex has been associated with a better response to PEG-IFN-alpha ^99, 100^, although this effect is not consistent across all studies ^101^. Likewise, a female advantage is observed for nucleos(t)ide analogue treatment: in a study of >2000 HBV patients starting treatment with entecavir, earlier virologic response was observed in treatment-naïve females compared to males ^102^. In HIV/HBV coinfected patients starting antiviral therapy, females have a higher rate of functional cure within two years (OR for HBsAg clearance in males 0.54 compared to females as reference group) ^103^ and male sex is reported in association with failure to suppress HBV viraemia ^104^.

## 5. Sex to inform HBV interventions

### 5.1 Sex to inform stratification for anti-viral therapy

Traditionally, HBV infection has been classified based on serology, VL, and markers of liver damage (such as liver enzyme levels, imaging scores and/or liver biopsy) ^105^. Guidelines for nucleoside/nucleotide analogue (NA) therapy, based on these biomarkers together with age and sex, typically consider ~1 in 4 individuals with CHB treatment-eligible, although this varies between populations ^106^. However, there is increasing recognition that current classification systems over-simplify liver disease by applying a traditional paradigm of linear progression, and that untreated individuals have a risk of progressive liver disease and HCC, and may also be at risk of transmitting infection ^106– 109^.

An increased understanding of the role of sex as a risk factor for disease could allow refinement of treatment algorithms, for example lowering the threshold for treatment of males and post-menopausal females, such that more individuals are treatment eligible. Treatment with NA agents such as tenofovir (typically prescribed as tenofovir disoproxil fumarate, TDF) is cheap, safe and effective in suppressing viraemia. Expanding CHB treatment is a key intervention to improve progress towards elimination goals ^76, 110^ by offering therapy to those at greatest risk of long-term disease ^111^ as well as switching off transmission. Enhanced approaches to risk stratification will also benefit from an improved understanding of the interactions between sex and other attributes of the host (e.g. age, co-morbidity, gravidity, ethnicity) and virus (VL, genotype), which are yet to be fully resolved.

Sex and gender are likely to be significant factors in receipt of treatment. Universal health coverage (UHC) is included in SDG targets, setting a mandate for access to healthcare irrespective of demographic factors including sex, age, and ability to pay. For CHB, sex disaggregated data are currently insufficient to estimate the proportion of the untreated CHB population who are male vs. female ^112^. However, treatment coverage may be influenced by biology and natural history of infection, in which higher VL and more advanced disease in males make them more likely to meet eligibility criteria than age-matched females.

Access to screening and engagement with care are also dependent on many other societal and infrastructure considerations, including education, beliefs and behaviour, and the structure of health-care services (discussed further in section 6). This topic has been explored for HIV, in which a higher proportion of women access treatment, have better immunological responses to treatment, and are retained in long-term follow-up ^113, 114^, but no such data are available for HBV infection.

### 5.2 Sex to inform HCC risk assessment and therapy

Risk scores have been proposed to predict HCC risk through measurement of non-invasive parameters such as VL, platelet count, and liver enzyme levels ^115^, with risks of HCC increasing with VL >2000 IU/ml ^116^. A recent systematic review and metanalysis evaluated the performance of these scores ^117^. 12 of the 14 scores used sex as part of the algorithm, but the use of sex as a simple categorical variable may be over simplistic. For example, the REACH-B score assigns a value of 2 to males and 0 to females ^118^, without considering enhanced risks in older women. It should also be noted that there was a male predominance in the validation cohorts which may affect the assessment of relative risk between the sexes.

As evidence emerges for the role of sexual dimorphism in metabolic and malignant disease, this may shed light on new therapeutic approaches using hormonal therapy or blockade ^119, 120^. Androgen receptor (AR) blockade has thus far not shown therapeutic benefits for HCC ^79, 121, 122^, but as we improve understanding of the biological pathways involved in driving cancer, this strategy warrants further exploration.

### 5.3 Interplay between age and sex

Alongside sex, another factor of considerable importance is age (first noted by Blumberg in his 1972 study, in which sexual dimorphism was most striking in the youngest groups ^15^). Given the strong observed effect of sex, it is to be expected that the natural history of CHB may change throughout life due to changes in the levels of sex steroids. For females, menarche, pregnancy, and menopause are therefore of particular relevance.

Earlier menarche has been correlated with earlier HBeAg seroconversion and a faster rate of HBsAg titre decline ^123^. In contrast, early menarche has elsewhere been shown to correlate with an increased risk of HCC in HBsAg carriers ^124^. A protective effect of early puberty has also been described in a small cohort of males ^125^, perhaps counter-intuitively given that increased testosterone is otherwise identified as a risk factor. This reflects puberty as a period of complex immunological change, and highlights the need for further study. Postmenopausal status has been shown to reduce, or even remove, the protective effect of female sex. A multicentre cross-sectional study in China, involving 17,408 patients with CHB, found that the prevalence of cirrhosis increased at a faster rate after the age of 50 in females than in males ^126^, and likewise sex differences diminished among older adults in a large Taiwanese cohort ^32^. The risk of liver fibrosis in CHB has similarly been reported as comparable between post-menopausal females and age-matched males ^127^. The loss of protection associated with menopause can be mitigated by hormone replacement therapy (HRT), which has a protective effect proportional to the duration of treatment ^124^. Similar observations have been made in other chronic infections: in HCV infection, the risk of liver disease is lower in pre-menopausal females and accelerates to match that of males when the protective effect of oestrogen exposure is lost ^128^; in HIV the TLR-7 response and IFN production in women are dampened after the menopause, and restored by oestrogen replacement (reviewed in 129).

Together these data confirm that oestrogen has a protective and dose-dependent effect on the course and characteristics of CHB, which may vary depending on the life history of the individual female. The nuance of hormonal influence in the natural history of CHB in females requires more explicit study, but this is an area in which enhanced understanding could impact on simple interventions such as enhanced surveillance, and antiviral and/or HRT prescription for post-menopausal women.

### 5.4 Maternal and child health

During pregnancy, there is a general tolerization of the immune system, with alterations in the Th1/Th2 ratio (with downregulation of Th1 immunity to avoid rejection of the foetus), such that Th1 cytokines (IFN-gamma and IL-2) are reduced, and there is a relative increase in Th2 cytokines (such as IL-4). These changes may have an impact on HBV infection in the mother ^130, 131^: during pregnancy, both increases and decreases in HBV DNA VL have been reported compared to non-pregnant women (reviewed in 132), while ALT flares are well recognised both during pregnancy and post-partum. Post-partum flares are mediated potentially by changes in the immune response (to re-set the non-pregnant Th1/Th2 balance), but can also be related to withdrawal of short-term antiviral therapy instituted to prevent vertical transmission ^133– 135^. In the majority of cases, flares are classified as mild (e.g. ALT up to 5 times upper limit of normal, ULN) or moderate (ALT up to 10 times ULN), with no clear detriment to maternal and foetal outcomes ^136, 137^, and indeed potentially associated with clearance of HBsAg. Rarely, severe flares (ALT >10 times ULN) arise, which can be associated with fulminant hepatitis ^138^. Altered cytokine production and hepatic flares during pregnancy may stimulate clearance of HBeAg, and ultimately also loss of HBsAg (functional cure), although these events are uncommon ^131^. In a Turkish cohort, multigravid females had a higher seroprevalence of HBsAg than primigravidae ^139^, although this is difficult to interpret, as it may represent differences in exposure rates rather than a biological difference mediated by pregnancy. The risk of HCC in HBsAg positive women has also been inversely correlated with the number of pregnancies and the age of menopause ^140^.

HBV infections in children typically occur either at birth (vertical transmission), or in the first few years of life through horizontal transmission from close household contacts ^141^. Vaccination of infants at birth is a simple and effective method of reducing these early life infections, which account for most of the long-term burden of CHB. However, the effectiveness of birth vaccination is reduced when mothers have high HBV VL ^111^. Thus, although females typically have lower VL and a lower risk of long-term liver disease than males, provision of clinical care and interventions for women of child-bearing age and their infants (screening, monitoring, anti-viral therapy, and infant vaccination) are crucial public health interventions warranting sustained investment. The greater risk of persistent viral replication, cirrhosis, and HCC in individuals who have been vertically infected underlines the urgent need for diagnosis and intervention to prevent mother-to-child transmission ^142^.

Route of transmission, HBV genotype, and population setting may all interact with hormonal factors in mediating outcomes. For example, higher rates of vertical transmission in some settings (influenced by genotype) have been suggested to bring the male:female ratio closer to 1:1 in CHB ^32^.

## 6. The role of gender

The construct of gender, defined as ‘characteristics of women and men that are largely socially created’ ^143^ is important, accounting for social and behavioural factors. However, the term has often been used interchangeably with sex, and their influence can be difficult to disaggregate. The need to improving reporting of data representing both sex and gender is underpinned by Sex and Gender Equity in Research (‘SAGER’) guidelines, first published in 2016, to promote improved study design, analysis, and interpretation of clinical data ^144^. However, progress in this domain has been slow, with little improvement in reporting sex-disaggregated data among organisations reviewed between 2018 and 2021 ^145^.

Gender roles may influence care-seeking behaviour, particularly through services for women, who typically have more points of contact with health services as a result of child-bearing and childcare, although traditional responsibilities are increasingly changing. Access to healthcare, education and support networks frequently vary by gender, from early in childhood and throughout life. Access gaps may be particularly pertinent for adolescent boys and young adult men; based on the HIV literature, these groups may be least likely to seek out or engage with diagnostic and treatment services ^146, 147^. For HBV, the long-term risk of complications is highest in these young men due to the combination of pathophysiology of infection in males, combined with the difficulty of providing consistent, accessible and acceptable healthcare. Men may also be over-represented among people who inject drugs and the prison population, among whom risks of blood-borne virus exposure are higher.

Stigma is a barrier to diagnosis and intervention for HBV ^148^, with discrimination potentially further enhanced by gender and sexuality. Access to care for MSM, bisexual and trans-gender communities may be further complicated in countries where legislation prohibits same-sex relationships. HBV is endemic in MSM and transgender women (e.g. in Papua New Guinea ^149^ and Nigeria ^150^), but these groups may be hard to reach for screening, surveillance and treatment. High rates of drug and alcohol misuse may both increase the risk of exposure to infection with blood-borne viruses, and present barriers to interaction with healthcare services, as well as being associated with vaccine hesitancy in some populations ^151^.

## 7. Conclusion

**Figure 4.**
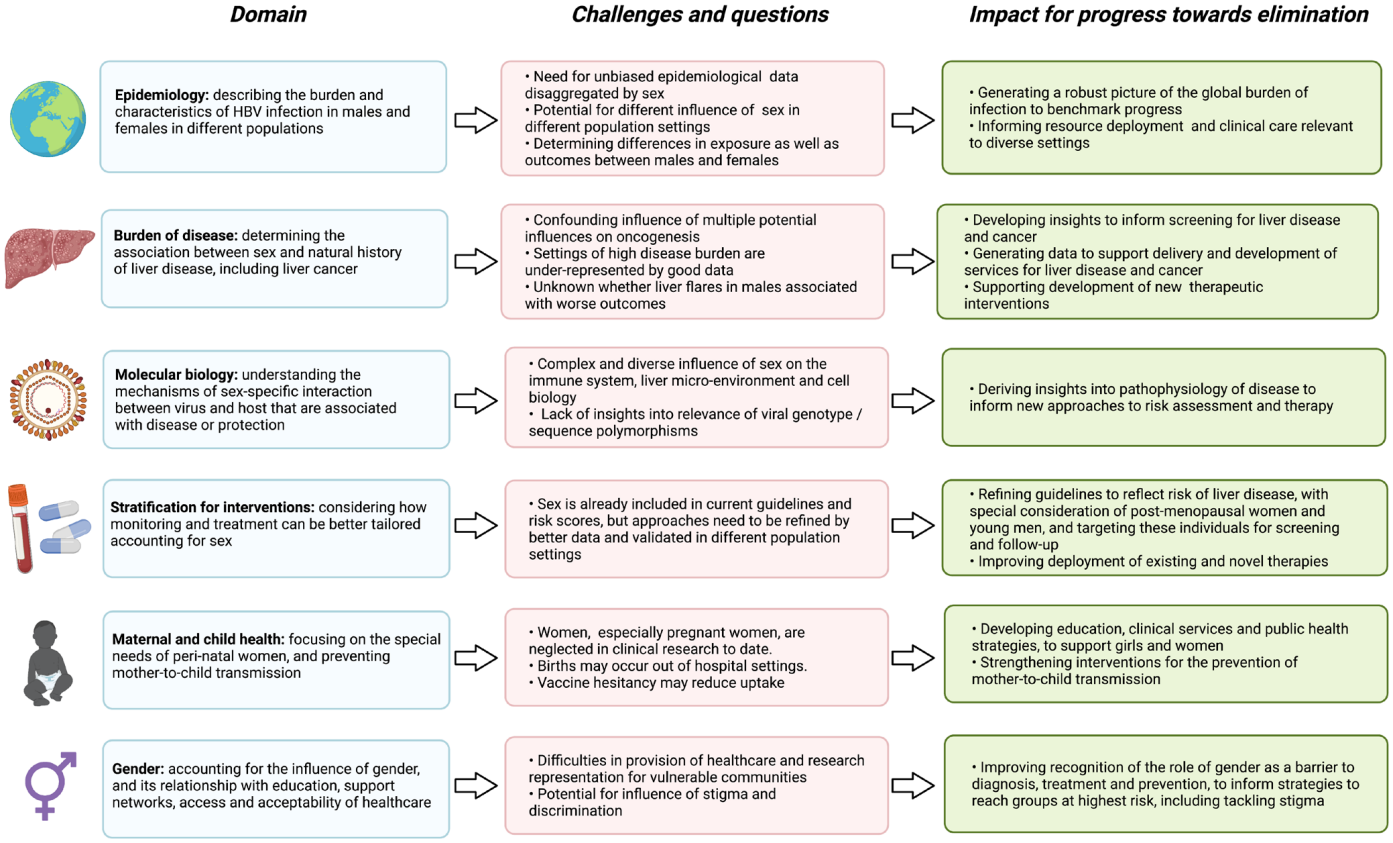
Summary of the interplay between sex and the outcomes of HBV infection, highlighting gaps and challenges in current understanding, and the impact for advances towards international elimination goals. Figure created with BioRender.com, with licence to publish.

Sex and gender have a fundamental impact on the course and outcomes of HBV infection ( Table 2), and developing insights in this domain has the potential to influence intervention ( Figure 4). The magnitude and nature of the sex effect vary between populations and with age, but - nearly forty years since the phenomenon of sexual dimorphism was first reported - have yet to be robustly quantified on a multiregional scale. Improving the reporting of sex and gender data is imperative for HBV, as stratification for treatment or surveillance by sex has the potential to improve outcomes for individuals, with associated population benefits in areas of high endemicity.

**Table 2.** Summary of the influence of sex on HBV infection.

Attribute of HBV infection	Impact of sex dimorphism
**Risk of exposure to infection**	• Higher in males than females
**Risk of development of chronic infection**	• Higher in males than females
**Risk of development of inflammatory/** **fibrotic liver disease**	• Higher in males than females • Female risk may increase post-menopause
**Development and outcomes of HCC**	• Higher incidence of HCC in males • Worse outcomes on treatment and shorter life expectancy in males
**Likelihood of receiving treatment**	• Higher proportion of males meet treatment eligibility criteria, based on higher VL and advanced disease • Behaviour and beliefs may prevent males from care seeking (also see gender, below)
**Representation by existing datasets**	• Likely to be better for males than for females, although certain female groups are better represented (e.g. pregnancy)
**Access to clinical care**	• May be more barriers for younger men • Women may be able to access screening and interventions through perinatal services • Special focus is required for post-menopausal women • Males are over-represented in certain risk groups for whom there are barriers in access to care (PWID, prison population)
**Likelihood of receiving treatment**	• May be higher in males based on higher VL and advanced disease (more likely to meet treatment thresholds), however also consider gender (see below)
**Interaction with gender**	• Access to diagnosis and care is influenced by gender-specific education, behaviour, beliefs, role-models and tailoring of health services. • May be particular barriers for MSM and trans-gender people

HCC – hepatocellular carcinoma, MSM – men who have sex with men, PWID – people who inject drugs, VL – viral load

Females with CHB currently suffer from a lack of specific research, that potentially disadvantages HBV elimination efforts as a whole. While women are at lower risk of chronic infection and liver disease, a focus on women’s health is nevertheless a fundamental aspiration for global health interventions for HBV, through reducing mother-to-child transmission, reducing the sex bias in data sets, and focusing attention on post-menopausal women who may be at increased risk of liver disease than younger females.

Enhancing an understanding of the mechanisms through which sex hormones mediate their influence can inform a better understanding of the pathophysiology of liver disease, with potentially important bearing on the use of existing interventions as well as informing the development of new therapies for HBV and the associated complication of HCC. Future research must focus on characterising the influence, impact and mechanisms of sexual dimorphism in HBV.

## Data availability

No underlying data are associated with this article.
