# Supplementary material for: Sexual Dimorphism in Chronic Hepatitis B Virus (HBV) Infection: Evidence to Inform Elimination Efforts
Source: Wellcome Open Res. 2022 May 11;7:32. Originally published 2022 Jan 31. [Version 3] doi: 10.12688/wellcomeopenres.17601.3 (PMC9520633; doi:10.12688/wellcomeopenres.17601.3)
Supplement: Peer review history

# Peer review: Reviewer response for version 2

**DOI:** 10.21956/wellcomeopenres.19779.r50133

**Published:** 2022-04-27

**Authors:** Yuchen Xia

**Affiliations:** 1 State Key Laboratory of Virology and Hubei Province Key Laboratory of Allergy and Immunology, Institute of Medical Virology, School of Basic Medical Sciences, Wuhan University, Wuhan, China

In Figure 3, HNF4-α should be HNF4-α. The panel label (A, B, according to legend) is missing.

Is the review written in accessible language?

Yes

Are all factual statements correct and adequately supported by citations?

Yes

Are the conclusions drawn appropriate in the context of the current research literature?

Yes

Is the topic of the review discussed comprehensively in the context of the current literature?

Yes

Reviewer Expertise:

hepatitis B

I confirm that I have read this submission and believe that I have an appropriate level of expertise to confirm that it is of an acceptable scientific standard.

# Peer review: Reviewer response for version 2

**DOI:** 10.21956/wellcomeopenres.19779.r50134

**Published:** 2022-04-27

**Authors:** Andri Rauch

**Affiliations:** 1 Department of Infectious Diseases, Inselspital, Bern University Hospital, University of Bern, Bern, Switzerland

I thank the authors for the revision which further improved this important review and recommend to accept the current version of this manuscript.

Is the review written in accessible language?

Yes

Are all factual statements correct and adequately supported by citations?

Yes

Are the conclusions drawn appropriate in the context of the current research literature?

Yes

Is the topic of the review discussed comprehensively in the context of the current literature?

Yes

Reviewer Expertise:

Infectious diseases; HIV; viral hepatitis

I confirm that I have read this submission and believe that I have an appropriate level of expertise to confirm that it is of an acceptable scientific standard.

# Peer review: Reviewer response for version 1

**DOI:** 10.21956/wellcomeopenres.19463.r49465

**Published:** 2022-04-11

**Authors:** Andri Rauch

**Affiliations:** 1 Department of Infectious Diseases, Inselspital, Bern University Hospital, University of Bern, Bern, Switzerland

Brown and colleagues review the role of sexual dimorphism in chronic HBV infection. The review is comprehensive and very well written and presented. The tables and figures provide an excellent overview of the current knowledge in the field. The review includes very useful information on sexual dimorphism with regard to epidemiology, underlying mechanisms, clinical outcomes and treatment.

 I have just two suggestions, which could supplement the current review:

- The studies presented in Table 1 do not include studies from Africa, and there is overall little information in the review in this context. It would be important to discuss this in more detail, particularly in the context of perinatal transmission where the reasons for sexual dimorphism might differ from the situation in sexual or blood-borne transmissions.
- The section on behavioural factors could be expanded with regard to sexual risk factors which could alter transmission rates, and factors which accelerate liver disease (e.g. alcohol and drug use). This topic is mentioned in section “ *6 The role of gender” *but could be expanded given the potentially important role of behavioral factors.

Is the review written in accessible language?

Yes

Are all factual statements correct and adequately supported by citations?

Yes

Are the conclusions drawn appropriate in the context of the current research literature?

Yes

Is the topic of the review discussed comprehensively in the context of the current literature?

Yes

Reviewer Expertise:

Infectious diseases; HIV; viral hepatitis

I confirm that I have read this submission and believe that I have an appropriate level of expertise to confirm that it is of an acceptable scientific standard.

# Response

**Published:** 2022-04-14

**Authors:** Philippa Matthews

**Affiliations:** Nuffield Department of Medicine, Peter Medawar Building for Pathogen Research, University of Oxford, UK

***Author response:*** *we are grateful for positive feedback overall; thank you.*

**Reviewer comment:** I have just two suggestions, which could supplement the current review. The studies presented in Table 1 do not include studies from Africa, and there is overall little information in the review in this context. It would be important to discuss this in more detail, particularly in the context of perinatal transmission where the reasons for sexual dimorphism might differ from the situation in sexual or blood-borne transmissions.

***Author response:*** *thank you for this important feedback. We agree that Africa is under-represented. Although small studies have been published from individual centres, these rarely provide line-level data that would allow disaggregation by sex, and thus robust, representative data for HBV infection according to sex are not available for any African populations (to the best of our knowledge). Indeed, we have ourselves set out to undertake a meta-analysis for African data to address this question, and found we were unable to do so.*

*We have amended the text as follows to draw specific attention to this gap in global data: *

- *‘* *It is notable that the data included in Table 1 do not include any representation of African populations, as a result of the lack of robust metadata available from the African continent* *.’ *
- *We have retained existing citations to African metanalyses, but providing a context that links back to the overall lack of data: ‘For example, large scale HBV metanalyses of HBV prevalence from Ethiopia and Burkina Faso were unable to determine a sex ratio (25, 26).’ *
- *Recognising the importance of this point, we have also added to the conclusion: ‘Some regions and populations are yet to be represented in epidemiological data; at present there are no data informing meta-analysis representing the WHO African region.’*

*Regarding the point about perinatal transmission, we have expanded section 5.4 (maternal and child health) to incorporate this feedback, with three additions to the existing text, as follows:*

- *‘HBV infections in children typically occur either at birth (vertical transmission), or in the first few years of life through horizontal transmission from close household contacts (141). The sex of the child may alter susceptibility, as evidenced by work in the HIV field, in which female fetuses have a 1.5-2-fold increased susceptibility to in utero infection compared to males (10); although there are no such data for HBV, this finding is a biological precedent for the impact of sexual dimorphism from the earliest days of life.’*
- *‘The greater risk of persistent viral replication, cirrhosis, and HCC in individuals who have been vertically infected underlines the urgent need for diagnosis and intervention to prevent mother-to-child transmission and other early-life acquisition events (142). Improved epidemiological data regarding HBV infections occurring in utero, in infancy and during childhood, stratified according to maternal characteristics and the sex of the offspring, could provide important insights into the biology of HBV transmission.’*
- *‘Regional studies are particularly needed to provide an evidence base for high endemicity populations in the African and Asian subcontinents.’*

**Reviewer comment:** The section on behavioural factors could be expanded with regard to sexual risk factors which could alter transmission rates, and factors which accelerate liver disease (e.g. alcohol and drug use). This topic is mentioned in section “6 The role of gender” but could be expanded given the potentially important role of behavioral factors.

***Author response:*** *thank you for this important feedback. As suggested, we have expanded this section, and also included some additional references. New text reads as follows:*

- *Women are at increased risk of BBV transmission where their access to education is limited, and in the absence of reliable access to sexual and reproductive health services.*
- *HBV is endemic in some communities of MSM and transgender women (e.g. in Papua New* *Guinea and Nigeria ^^), and providing reliable healthcare for these groups is challenging. Access to care for MSM, bisexual and transgender communities may be further complicated in countries where legislation prohibits same-sex relationships. A study in Spain to determine the incidence of acute HBV infection in people living with HIV reported that this was higher in males, particularly in MSM , and HBV infection has also been associated with risky sexual practices and transactional sex. High rates of drug and alcohol misuse increase the risk of exposure to infection with BBV, and accelerate liver disease in those with HBV infection, create barriers to interaction with healthcare services, and are associated with vaccine hesitancy in some populations. Stigma is a barrier to diagnosis and intervention for HBV,* *with discrimination potentially further enhanced by gender and sexuality. Extreme marginalisation in some of the groups at high risk of HBV infection is a significant barrier to education, diagnosis, treatment and prevention. *
- *We have also expanded the final row of Table 2 (interaction with gender) to include ‘* *Risk factors overlap with alcohol excess, substance abuse, incarceration, transactional sex.'*

*New references to support the additional text include PMID: 34527207, 34229613, 34112187, 32738575 and 35249671.*

# Peer review: Reviewer response for version 1

**DOI:** 10.21956/wellcomeopenres.19463.r49394

**Published:** 2022-04-11

**Authors:** Yuchen Xia

**Affiliations:** 1 State Key Laboratory of Virology and Hubei Province Key Laboratory of Allergy and Immunology, Institute of Medical Virology, School of Basic Medical Sciences, Wuhan University, Wuhan, China

Chronic hepatitis B virus infection (HBV) creates a global health burden, with about 240 million people persistently infected. It is also the major cause of hepatocellular carcinoma (HCC). Gender disparity is known to play a role in HBV infection and the development of HBV related-HCC. This review provides a comprehensive summary of current knowledge about the sex differences in chronic HBV infection from virus infection rate, morbidity and mortality, immunity, treatment response and age, etc. Strengths of this manuscript include its broad scope, extensive referencing of the primary literature, and balance in how it addresses the unknowns in the field. It will be a valuable addition to the scientific literature.

 Weaknesses overall are modest. They include:

1. In 3.2 part, the HCC induced by carcinogens such as DEN does not fit the topic well.
2. In 4.1 part, this part did not mention vaccination, the subtitle was not appropriate.
3. In 4.3 part, reference ‘18’ is a review. The original literature should be cited. In addition, reference 80 regarding ERα and HNF-4α was not the right one.
4. In table 2, ‘Likelihood of receiving treatment’ is repeated twice.
5. In 4.4 part, this section mentioned the effect of sex on treatment response. It should be moved to section 5.1.
6. In figure 3 legend, hepatocyte nuclear factor 4-alpha abbreviation should be HNF4-α. Similar mistakes appeared in the text.

Is the review written in accessible language?

Yes

Are all factual statements correct and adequately supported by citations?

Yes

Are the conclusions drawn appropriate in the context of the current research literature?

Yes

Is the topic of the review discussed comprehensively in the context of the current literature?

Yes

Reviewer Expertise:

hepatitis B

I confirm that I have read this submission and believe that I have an appropriate level of expertise to confirm that it is of an acceptable scientific standard, however I have significant reservations, as outlined above.

# Response

**Published:** 2022-04-14

**Authors:** Philippa Matthews

**Affiliations:** Nuffield Department of Medicine, Peter Medawar Building for Pathogen Research, University of Oxford, UK

***Author response:*** *Thank you for the very positive feedback overall.*

***Reviewer comment: *** In 3.2 part, the HCC induced by carcinogens such as DEN does not fit the topic well.

***Author response:*** *our intention in this section is to highlight the male susceptibility to liver cancer (both driven by HBV and by other aetiologic agents). In order to set the context more clearly, we have readjusted the text, such that the section in question now opens by providing the rationale for citing animal models, acknowledges that oncogenesis in males is irrespective of aetiology, and underlines that DEN is cited just as one example of a carcinogen that can be introduced into a model system: The text now reads as follows: ‘These observations in humans are corroborated by evidence from animal models that explore sex-related susceptibility to the development and progression of liver cancer, irrespective of aetiology. For example, following exposure to high dose carcinogens (such as diethylnitrosamine (DEN)), liver inflammation and proliferation are more pronounced in male mice (52), and males exhibit a higher rate of tumorigenesis (53), with cancers that progress more rapidly.’*

***Reviewer comment: *** In 4.1 part, this part did not mention vaccination, the subtitle was not appropriate.

***Author response:*** *the original text in this section briefly mentioned vaccination in saying that females have enhanced vaccine responses, and referring the reader to a later section of the article. However, acknowledging that vaccination is not the primary topic addressed in Section 4.1, we have taken this out of the section subtitle. The simplified subtitle now reads ‘Immunological differences between males and females causing differential outcomes of infection’. We have also expanded the link to information about vaccination in a later section, by pointing the reader specifically to the relevant text as follows: ‘(discussed in further detail in section 4.2 below)’.*

***Reviewer comment: *** In 4.3 part, reference ‘18’ is a review. The original literature should be cited. In addition, reference 80 regarding ERα and HNF-4α was not the right one.

***Author response:*** *We have amended to clarify that reference 18 is a review, so our text now reads as follows: ‘The viral genome also has a direct influence through sex hormone response elements: (previously reviewed (18), and summarised in Figure 3)’. We completely agree that reference to the primary literature is important, and these references are cited throughout section 4.3 and in Figure 3 legend (references numbered 78-87). We have double-checked our citation of Reference 80 (Wang SH, et al.: Estrogen receptor α represses transcription of HBV genes via interaction with hepatocyte nuclear factor 4α. Gastroenterology. 2012;142(4):989–998.e4. 22240483 10.1053/j.gastro.2011.12.045) and this appears to be correct – but if the reviewer can explain further any concerns about an error here, we will be happy to amend.*

***Reviewer comment: *** In table 2, ‘Likelihood of receiving treatment’ is repeated twice.

***Author response:*** *thank you for spotting this error; we have removed the duplicated row.*

***Reviewer comment: *** In 4.4 part, this section mentioned the effect of sex on treatment response. It should be moved to section 5.1.

***Author response:*** *in the original version of the article, we had two sections about sex and treatment, the first (section 4.4) was about the biology of treatment response, and the second (section 5.1) considered potential practical approaches to changing treatment guidelines. In response to this feedback, we have amalgamated to combine into a single section (now all within section 5.1).*

***Reviewer comment: *** In figure 3 legend, hepatocyte nuclear factor 4-alpha abbreviation should be HNF4-α. Similar mistakes appeared in the text.

***Author response:*** *thank you for highlighting this; we have adjusted the figure legend and text accordingly. We have also corrected Erα to ER-α.*
